# *Nigella sativa* Relieves the Altered Insulin Receptor Signaling in Streptozotocin-Induced Diabetic Rats Fed with a High-Fat Diet

**DOI:** 10.1155/2016/2492107

**Published:** 2016-08-04

**Authors:** Mahmoud Balbaa, Marwa El-Zeftawy, Doaa Ghareeb, Nabil Taha, Abdel Wahab Mandour

**Affiliations:** ^1^Biochemistry Department, Faculty of Science, Alexandria University, Alexandria 21511, Egypt; ^2^Biochemistry Department, Faculty of Veterinary Medicine, Alexandria University, Alexandria, Egypt

## Abstract

The black cumin (*Nigella sativa*) “NS” or the black seeds have many pharmacological activities such as antioxidant, anticarcinogenic, antihypertensive, and antidiabetic properties. In this work, streptozotocin-induced diabetic rats fed with a high-fat diet were treated daily with NS oil (NSO) in order to study the effect on the blood glucose, lipid profile, oxidative stress parameters, and the gene expression of some insulin receptor-induced signaling molecules. This treatment was combined also with some drugs (metformin and glimepiride) and the insulin receptor inhibitor I-OMe-AG538. The administration of NSO significantly induced the gene expression of insulin receptor compared to rats that did not receive NSO. Also, it upregulated the expression of insulin-like growth factor-1 and phosphoinositide-3 kinase, whereas the expression of ADAM-17 was downregulated. The expression of ADAM-17 is corroborated by the analysis of TIMP-3 content. In addition, the NSO significantly reduced blood glucose level, components of the lipid profile, oxidative stress parameters, serum insulin/insulin receptor ratio, and the tumor necrosis factor-*α*, confirming that NSO has an antidiabetic activity. Thus, the daily NSO treatment in our rat model indicates that NSO has a potential in the management of diabetes as well as improvement of insulin-induced signaling.

## 1. Introduction

Diabetes mellitus is the most common metabolic disorder characterized by hyperglycemia that results from defects in insulin secretion or action or both, which affect carbohydrate, lipid, protein, and nucleic acid metabolism [1]. It can be divided primarily into two types: type I or insulin dependent diabetes mellitus and type II or noninsulin dependent diabetes mellitus [2]. Type I diabetes mellitus is an autoimmune disease characterized by local inflammatory reaction in and around islets that is followed by selective destruction of insulin secreting *β*-cell and it occurs mainly in childhood [3]. On the other hand, type II is mainly due to hereditary factors, affluent lifestyles, and obesity. It is characterized by peripheral insulin resistance and impaired insulin secretion [2]. Moreover, the primary hormone involved in control of blood glucose is insulin. Once insulin is produced in the blood, it controls the glucose homeostasis by stimulating the clearance of glucose into skeletal muscle and, to a lesser degree, liver and adipose tissue. In muscle and adipocytes, insulin-stimulated glucose uptake is achieved by the translocation of the insulin-sensitive glucose transporter from intracellular storage vesicles to the cell surface [4]. Insulin binds to IR which is a heterotetramer protein consisting of two extra cellular *α*-subunits and two transmembrane *β*-subunits held together by disulfide bonds [5]. However, several classes of hypoglycemic agents have been developed over the years to use in treatment of diabetes mellitus. Sulphonylureas and biguanide are two of the oldest classes of oral hypoglycemic agents. GLI is one of the sulphonylureas classes and it reduces the hyperglycemia by enhancing insulin secretion, while MET is one of the biguanide classes and acts to improve insulin sensitivity and suppress hepatic glucose output [6].

Naturally occurring phytochemicals are relatively nontoxic, inexpensive, and available in an ingestive form. Therefore, they are commonly used to prevent morbidity and mortality from chronic diseases in countries [7]. Among the beneficial medicinal plants* Nigella sativa* (NS), a dicotyledon of the Ranunculaceae family, is an amazing herb with a rich historical and religious background. The seeds of this plant are called black cumin or black seeds. The active principle of the volatile oil of NS seeds is called thymoquinone (2-isopropyl-5-methyl-1,4-benzoquinone). NS seeds possess several pharmacological activities such as neuroprotective [8], nephroprotective [9], antimutagenic [10], anticarcinogenic [11], and anticonvulsant activities [12]. Moreover, NS is known for its hypotensive [13], hepatoprotective [14], and immunomodulatory effects [15]. Thus, the objective of the present study was to throw the light on the effect of NSO, MET, and GLI monotherapies and NSO-MET or NSO-GLI on IR to investigate the interaction between herbs and drugs in an appropriate animal model that is analogous to the human type II diabetes mellitus. This was achieved through feeding animals with HFD followed by an injection of a low dose of STZ injection. To understand the effect of NSO on IR-induced signaling, the action of I-OMe-AG538 as an IRI on NSO-treated diabetic model of rats was investigated.

## 2. Materials and Methods

### 2.1. Materials

Kits and reagents for the assay of BGL, TC, TG, HDL-c, and protein were obtained from NS Biotec, Egypt. 2x Taq master mix, DNA Ladder, RNase-free water, *β*-actin, and the primer sequences of insulin, IGF-1, ADAM-17, and PI3K were obtained from Vivantis International Company, Germany. IR *β*-antibody (C18C4) and mouse IgG antibody were purchased from Novus Biologicals, USA. Maxime RT Premix Kit was purchased from INtRon Biotechnology, Korea. Rat TIMP-3 ELISA kit (ER1384) was obtained from Wuhan Fine Biological Technology Company, Wuhan, Hubei, China. I-OMe-AG538 and STZ were purchased from Sigma-Aldrich, USA. MET and GLI were obtained from the local markets. Other reagents were obtained with high grade. The composition and preparation of HFD were described in Table 1. HFD contained 58% fat, 25% protein, and 17% carbohydrate as a percentage of total calories. HFD was freshly made up on a weekly basis and stored at 4°C. NPD contained 11% fat, 16% protein, and 15% carbohydrate [16, 17].

### 2.2. Rats

Male albino Sprague-Dawley rats (*Rattus norvegicus*), of body weight 110–150 g, were obtained from the animal house of Faculty of Medicine, Alexandria University, Egypt. Rats were housed in polycarbonate cages in groups of 5 rats per cage. They were kept under conventional conditions of temperature and humidity with a 12 h photoperiod. Food and water were supplied* ad libitum*. All the experimental procedures were conducted according to the animal protocols approved by the Ethics Committee of Faculty of Science, Alexandria University, Egypt. Noninsulin dependent diabetes was induced in rats by HFD dietary manipulation for 2 weeks as previously described [16, 17]. After 2 weeks of HFD, overnight fasted rats were injected intraperitoneally with a low dose of STZ (35 mg/kg Bwt) dissolved in 0.1 M sodium citrate buffer, pH 4.5 [18]. The rats had free access to food and water and were given 5% glucose in their drinking water for the first 24 h to counter any initial hypoglycemia. The development of diabetes was confirmed after 72 h of STZ injection. The animals with BGL more than 250 mg/dL were considered diabetic. Out of 90 rats subjected for diabetes induction, 7 rats died before grouping and three rats were omitted from the study because of subdiabetic condition (108 mg/dL, 112 mg/dL, and 122 mg/dL). Both GLI (0.8 mg/kg Bwt) and MET (100 mg/kg Bwt) were used as reference drugs.

### 2.3. Rat Groups

The rats were randomly divided into three main groups, which were subdivided into subgroups. Group 1 represented nondiabetic untreated rats that were healthy, free from any disease, fed on NPD, and injected i/p by 0.1 M sodium citrate buffer, pH 4.5, or received daily a normal saline by intragastric tube at time of treatment of other groups [19]. Group 2 was fed HFD for 2 weeks and then injected with 35 mg/kg STZ. NSO, GLI, and MET-administrated subgroups of either group 1 or group 2 rats received daily 100 mg NSO in 2 mL 10% DMSO/kg Bwt, 0.8 mg/kg Bwt GLI, and 100 mg/kg Bwt MET by intragastric tube for 21 days. Both GLI and MET were dissolved in ddH_2_O before administrating to rats. Moreover, 2 mL of 10% DMSO/kg was administrated orally to another subgroup of rats for 21 days, whereas it was considered a vehicle for NSO during its extraction. Also, a combination of NSO and MET (NSO-MET) or NSO and GLI (NSO-GLI) was administrated to the other two subgroups either to healthy or diabetic groups of rats. Group 3 included the IRI I-OMe-AG538-treated subgroups. I-OMe-AG538 was dissolved in DMSO (10 mg/mL) and stored as a stock solution at −20°C [20]. This stock solution was diluted to 1.0 mg/mL in PBS and its LD_50_ was calculated according to the equation: Log LD_50_ (mg/kg) = 0.372 Log IC_50_ (*μ*g/mL) + 2.024 [21]. The subgroups of nondiabetic and diabetic rats were i/p injected with either 0.001 (IOMe 1) or 0.01 (IOMe 2) of I-OMe-AG538 LD_50_ for 21 days in the absence and presence of NSO administration. The selected dosage and duration of NSO treatment depend on its acute toxicity, behavioral changes, and mortality of rats.

### 2.4. Preparation of NSO

The seeds of NS were washed, dried, and finely powdered with a mortar and pestle. This was followed by adding 20 g of the powdered seeds to 400 mL of ddH_2_O and extraction was carried out by steam distillation. The distillation process was continued until about 200 mL of distillate was collected. The distillate was extracted three times with chloroform. Moisture was removed by anhydrous sodium sulphate and the resultant extract was evaporated using a 40°C water bath for the appearance of the volatile oil. An amount of 500 mg of the volatile oil was dissolved in 1 mL of DMSO, and then 9 mL of normal saline was added to yield a concentration of 50 mg volatile oil per 1 mL solution [22].

### 2.5. Biochemical Assays

Hepatic tissues from control and experimental groups were excised immediately and washed with ice-cold saline. Homogenization was carried out in 0.1 M sodium phosphate-buffer, pH 7.4 containing 0.9% sodium chloride. The homogenate was centrifuged at 5000 rpm for 10 min at 4°C to remove cellular debris and supernatant was used for biochemical analysis. BGL was measured at day 3 and every 10 days after STZ, NSO, GLI, MET, NSO-MET, and NSO-GLI treatments, respectively. Glucose levels in all groups were measured by a glucose assay kit that is dependent on the assay by glucose oxidase-peroxidase test [23]. The total protein was determined by a spectrophotometric assay [24]. Serum insulin of all groups was assayed by Sigma-Aldrich insulin ELISA kit [25]. The insulin resistance was evaluated by calculating the homeostasis model assessment estimate of insulin resistance (HOMA-IR) and HOMA-*β* as previously described [26]. TC was determined by cholesterol oxidase-peroxidase (CHOD-PAP test) [27]. TG was determined spectrophotometrically according to the previous methods [28]. HDL-c was determined by HDL-precipitating reagent [29] using a commercially available kit. LDL-c levels were calculated by using a specific formula [30]. The atherogenic index (AI) and the antiatherogenic index (AAI) were then calculated [31]. The level of TBARS was determined in serum and liver homogenate as previously described [32], whereas one molecule of malondialdehyde (MDA) was reacted with two molecules of thiobarbituric acid with the production of pink pigment with a maximum absorbance of 540 nm [33]. The level of NO was determined according the resulting azo dye, which has a bright-reddish-purple color with a maximum absorbance of 550 nm [34]. IR-*β* submit and TNF-*α* were determined by ELISA kit (Sun Red, England) in serum and/or liver homogenate. TIMP-3 was determined in liver homogenate by ELISA kit. These assays employ the quantitative sandwich enzyme immunoassay technique. A monoclonal antibody to rat IR-*β* submit, TNF-*α*, and TIMP-3 was precoated onto a microplate.

### 2.6. Primers PCR

Primers were designed using the known sequences for the respective genes (Table 2). Programs are given as denaturation temperature/denaturation times/annealing temperature/annealing times/extension temperature/extension times/number of cycles. The primers were run on MiniCycler (Eppendorf, Labcaire, Germany).

### 2.7. RNA Isolation and PCR

At the end of the treatments, overnight fasted rats were sacrificed under anesthesia. The liver was separated, washed with ice-cold normal saline, treated with liquid nitrogen, and stored at −80°C for total RNA isolation. Total RNA was extracted by using Total RNA Extraction Kit. Briefly, 10–100 mg of liver was homogenized in 250 *μ*L PBS, DEPC-treated H_2_O, and 750 *μ*L Easy-RED*™* solution and processed according to kit manufacturer's instructions. After that the concentration of total RNA was measured by spectrophotometer. cDNA was produced with 1 *μ*g of RNA as a template (Maxime RT Premix Kit and 2X Taq master mix were used). For gene expression, the gene-specific primers were used and the programs (Table 2) were optimized for each primer pair and all programs started with a 2.0 min period at 94°C and ended with a 30 s extension at 72°C. The PCR products were run on 1.5% agarose gel. Gels were stained with ethidium bromide, visualized by 30 nm Ultraviolet Radiator, and photographic record was made. The optical density and the *μ*g content of bands were calculated by the UVIBAND MAX software program.

### 2.8. Histological Preparation of Liver Tissue

The liver of two rats from each group was excised and immediately fixed at 10% neutral buffered formalin solution after washing with normal saline. The resultant fixed tissue samples were used for histopathological examination in the Histopathology Laboratory of Medical Technology Center, Alexandria University, using the routine procedures developed in the respective laboratories. The tissues were washed, dehydrated with alcohol, and cleared with xylene. Serial sections of 4 *μ*m thicknesses using a rotary microtome were deparaffinised with xylene and hydrated in descending grades of alcohol. The slides were then transferred to haematoxylin for 10 min, followed by rinsing with water, and later counterstained with eosin, rinsed with water, dehydrated with ascending grades of alcohol, cleared with xylene, and mounted. The slides were observed using a light microscope [35].

### 2.9. Statistical Analysis

All data are represented as mean ± SE. The statistical analysis was carried out by using the paired sample *t*-test (SPSS version 16). Values are considered statistically significant at *p* < 0.05.

## 3. Results

### 3.1. Body Weight and BGL

Twenty-one days posttreatment, body weight increased by 2.6-, 1.2-, 1.6-, 2.3-, and 2.1-fold when the diabetic rats were treated with NSO, MET, GLI, NSO-MET, and NSO-GLI, respectively. In the case of IRI injection, a decline of body weight was noticed (1.5-fold decrease) compared to control group. The treatment of such group with NSO showed a nonsignificant difference (*p* > 0.05) compared to IRI untreated rats. The treatment of diabetic rats with NSO for 21 days showed a significant decrease of BGL (142.76 ± 16.94 mg/dL) compared to 581.31 ± 36.31 mg/dL in diabetic rats. The treatment of diabetic rats with MET, GLI, NSO-MET, and NSO-GLI revealed significantly decreased levels of BGL (265.23 ± 20.67, 359.94 ± 17.29, 112.46 ± 3.94, and 106.62 ± 3.79 mg/dL, resp.) compared to the diabetic rats (581.31 ± 36.31 mg/dL). I-OMe-AG538-injected rats showed a significant increase of BGL (224.62 ± 4.29 mg/dL) compared to control (75.53 ± 1.22 mg/dL). Also, the diabetic rats injected with I-OMe-AG538 showed a significant increase of BGL (224.62 ± 4.29 mg/dL) compared to control. The administration of NSO with I-OMe-AG538 showed a significant decrease in BGL (113.54 ± 1.83 mg/dL) compared to the diabetic rats injected with only I-OMe-AG538 (224.62 ± 4.29 mg/dL) (Table 3).

### 3.2. Lipid Profile and Atherogenic Parameters

**Figure 1:**
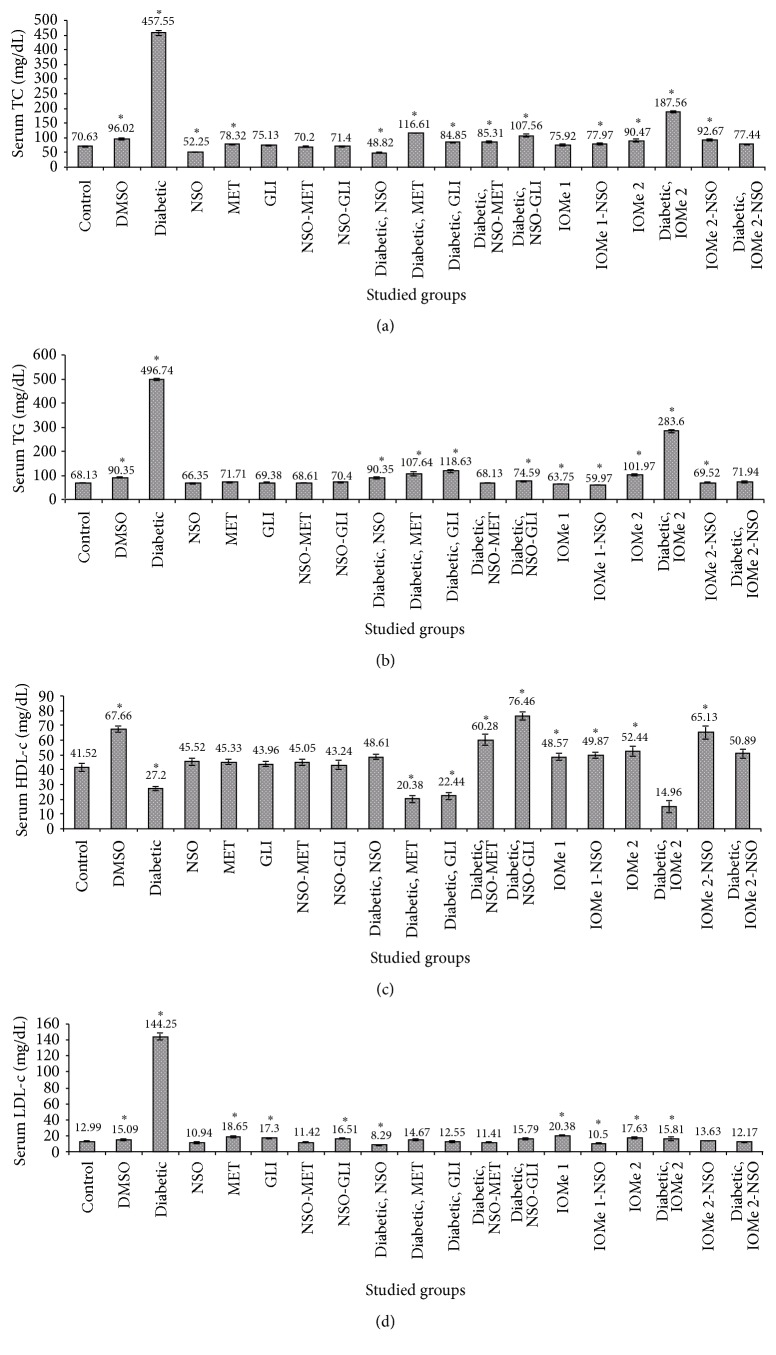
Lipid profile parameters. TC (a), TG (b), HDL-c, and LDL-c levels in rat sera after 21 days of the treatment with NSO, reference drugs, and I-OMe-AG538. (Data are expressed as mean ± SE. Significance is shown as (*∗*) at *p* < 0.05,  *n* = 10.)

There was a significant increase observed in TC and TG levels in the diabetic group in 21 days posttreatment. Increases of 3.0- and 4.5-fold for TC and increases of 6.5- and 7.3-fold for TG were observed, respectively, compared to normal control. Also, significant increases were noticed in both TC and TG in DMSO group (1.4- and 1.3-fold, resp.) compared to normal control (Figures 1(a) and 1(b)). The treatment of diabetic rats with NSO, MET, GLI, NSO-MET, and NSO-GLI decreased serum TC (9.4-, 3.9-, 5.4-, 5.4-, and 4.3-fold, resp.) compared to the diabetic group (Figure 1(a)). The same tendency was observed for serum TG (5.5-, 4.6-, 4.2-, 7.3-, and 6.7-fold, resp.) compared to diabetic group (Figure 1(b)). The injection of I-OMe-AG538 leads to serum TC elevation (1.3- and 1.5-fold, resp.) compared to normal control. The diabetic group injected I-OMe-AG538 showed 2.7- and 4.1-fold increase of serum TC and TG compared to normal control. Administration of NSO to I-OMe-AG538-injected group leads to 1.5-fold decrease of TG compared to I-OMe-AG538 group. The diabetic group injected I-OMe-AG538 and given NSO showed a significant 2.4- and 3.9-fold reduction in serum TC and TG, respectively (Figures 1(a) and 1(b)).

There was a significant decrease in HDL-c level after 21 days (1.5-fold). At the same time, the values of LDL-c level and AI were increased by 11.1- and 9.9-fold, respectively. AAI was decreased by 22.6-fold compared to normal control rats (Figure 1(c) and Table 3). HDL-c level was also decreased (1.6-fold) in DMSO-administrated group compared to normal control. Treatment of diabetic rats with NSO, MET, GLI, NSO-MET, and NSO-GLI leads to an increase of HDL-c level (1.8-, 1.3-, 1.2-, 2.2-, and 2.8-fold, resp.) compared to diabetic rats (Figure 1(d)). Significant reduction of LDL-c level was noticed in those treated groups (17.4-, 9.8-, 11.5-, 12.6-, and 9.1-fold, resp.) and reduction of AI (16.8-, 2.9-, 4.4-, 11.8-, and 11.9-fold, resp.). In reverse, a significant increase of AAI (3662.3-, 3.4-, 5.7-, 38.1-, and 38.9-fold, resp.) was observed compared to diabetic rats. On the other hand, treatment of diabetic rats with GLI leads to reduction of LDL-c and AI (11.5- and 4.4-fold, resp.) and an elevation of AAI (5.7-fold), but there was no a significant effect on HDL-c level compared to diabetic rats. The injection of rats I-OMe-AG538 showed an increase of HDL-c, LDL-c, and AAI (1.3-, 1.4-, and 21.8-fold, resp.) (Figure 1(c) and Table 3). Also, diabetic rats injected with I-OMe-AG538 showed decrease of HDL-c level (2.8-fold) and an increase of AI (7.4-fold) compared to normal control. I-OMe-AG538-administrated NSO showed an increase of HDL-c level and AAI (1.2- and 1.7-fold, resp.) and decrease LDL-c level (1.2-fold) compared to I-OMe-AG538 group. Also, diabetic group injected with I-OMe-AG538 and treated with NSO displayed increases of HDL-c and AAI (3.4- and 22.1-fold, resp.) and reduction of AI (8.25-fold) compared to I-OMe-AG538-injected diabetic group (Figure 1(d) and Table 3).

### 3.3. Insulin Resistance Parameters

Insulin concentration and HOMA-IR were increased (8.8- and 67.5-fold, resp.), while HOMA-*β* was decreased (4.7-fold) in the diabetic group compared to normal control. Significant levels of insulin (101.59 ± 5.78 *μ*IU/mL), HOMA-IR (145.82 ± 1.43 mg/dL), and HOMA-*β* (70.56 ± 5.12%) were displayed compared to control (11.59 ± 1.76 *μ*IU/mL, 2.16 ± 0.89 mg/dL, and 332.990 ± 9.08%, resp.). Also, the DMSO group displayed an elevation of insulin concentration and HOMA-IR (45.00 ± 0.68 *μ*IU/mL and 12.90 ± 0.76 mg/dL, resp.) and a nonsignificant reduction of HOMA-*β* (305.26 ± 13.01%) compared to normal control (Table 3). The treatment of diabetic rats with MET, GLI, NSO-MET, and NSO-GLI leads to a reduction of insulin level (1.9-, 1.4-, 1.1-, and 1.3-fold, resp.). Also, HOMA-IR was decreased (4.1-, 2.2-, 5.8-, and 7.3-fold, resp.) and HOMA-*β* was increased (1.4-, 1.3-, 9.4-, and 8.9-fold, resp.). NSO-treated group showed 1.3- and 8.2-fold increases in insulin level and HOMA-*β*, respectively, and 3.2-fold decrease in HOMA-IR compared to the diabetic group. I-OMe-AG538-treated nondiabetic and diabetic groups showed significant increases in insulin level, HOMA-IR, and HOMA-*β* compared to normal control. Administration of NSO to I-OMe-AG538 group leads to 7.8-, 10.3-, and 4-fold decreases in insulin level, HOMA-IR, and HOMA-*β*, respectively, compared to I-OMe-AG538-injected group. Also, administration of NSO to I-OMe-AG538-injected diabetic group leads to a reduction of HOMA-IR (2.0-fold) and an elevation of HOMA-*β* (3.1-fold) with nonsignificant effect on insulin level compared to I-OMe-AG538-injected diabetic group (Table 3).

### 3.4. Oxidative Stress Markers

**Figure 2:**
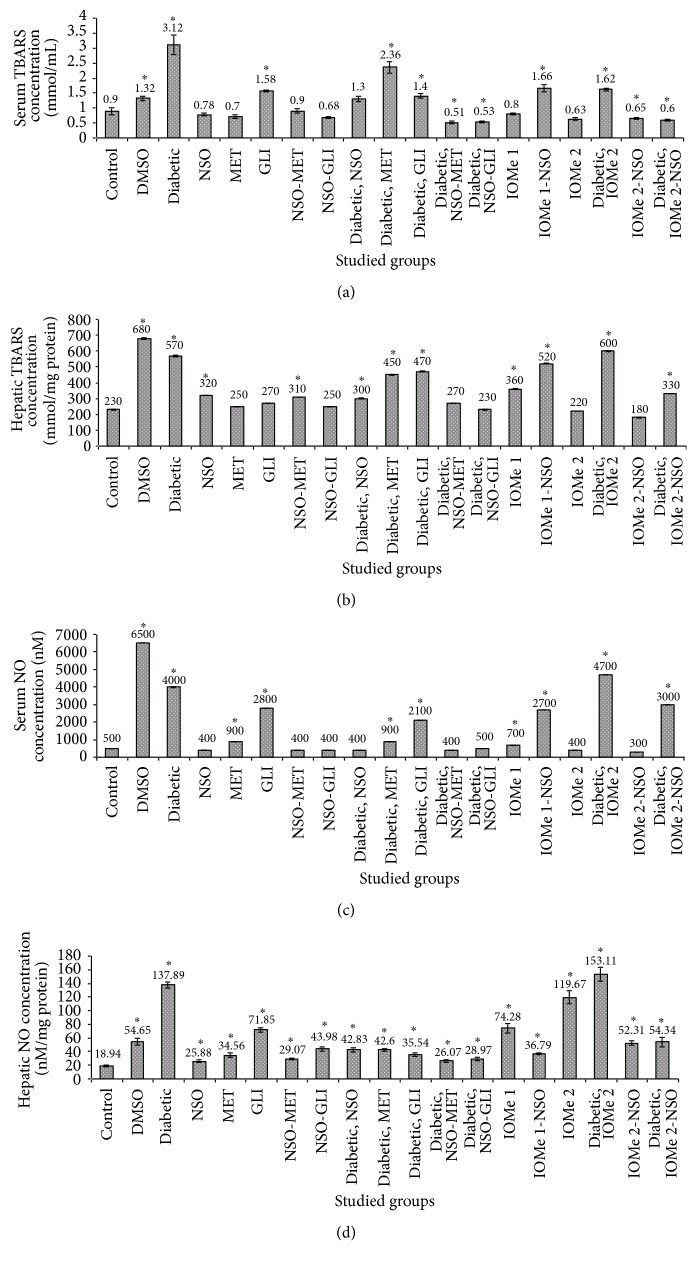
Oxidative stress markers. Serum (a) or hepatic (b) TBARS (a) and serum (c) or hepatic (d) NO levels after 21 days of the treatment with NSO, reference drugs, and I-OMe-AG538. (Data are expressed as mean ± SE. Significance is shown as (*∗*) at *p* < 0.05,  *n* = 10.)

Serum and hepatic TBARS and NO were increased in the diabetic group (3.5-, 2.5-, 8-, and 7.3-fold, resp.) and also in DMSO group (1.5-, 3.0-, 13-, and 2.9-fold, resp.) compared to normal control. Treatment of diabetic rats for 21 days with NSO, NSO-MET, and NSO-GLI leads to a decrease of serum TBARS (2.4-, 6.1-, and 5.9-fold, resp.) (Figure 2(a)), hepatic TBARS (1.9-, 2.1-, and 2.5-fold, resp.) (Figure 2(b)), serum NO (10.0-, 10.0-, and 8-fold, resp.) (Figure 2(c)), and hepatic NO (3.2-, 5.3-, and 4.8-fold, resp.) (Figure 2(d)). The treatment of diabetic rats after 21 days with MET ameliorated hepatic TBARS (1.3-fold) and serum and hepatic NO (4.4- and 3.2-fold, resp.). However, the treatment of diabetic rats for 21 days with GLI leads to a reduction of serum TBARS and hepatic NO (2.2- and 3.9-fold, resp.) compared to diabetic group. Injection of I-OMe-AG538 caused an elevation of hepatic NO (6.3-fold) compared to normal control. Also, I-OMe-AG538-injected diabetic group revealed an increase of serum and hepatic TBARS and NO (1.8-, 2.6-, 11.75-, and 8.1-fold, resp.) compared to normal control. Administration of NSO to I-OMe-AG538-injected nondiabetic group leads to a reduction of hepatic NO (2.3-fold). Also, administration of NSO to I-OMe-AG538-injected diabetic group showed a reduction of serum and hepatic TBARS and NO (1.9-, 1.8-, 1.6-, and 2.8-fold, resp.) (Figures 2(a)–2(d)).

### 3.5. Insulin Signaling Pathway Parameters

**Figure 3:**
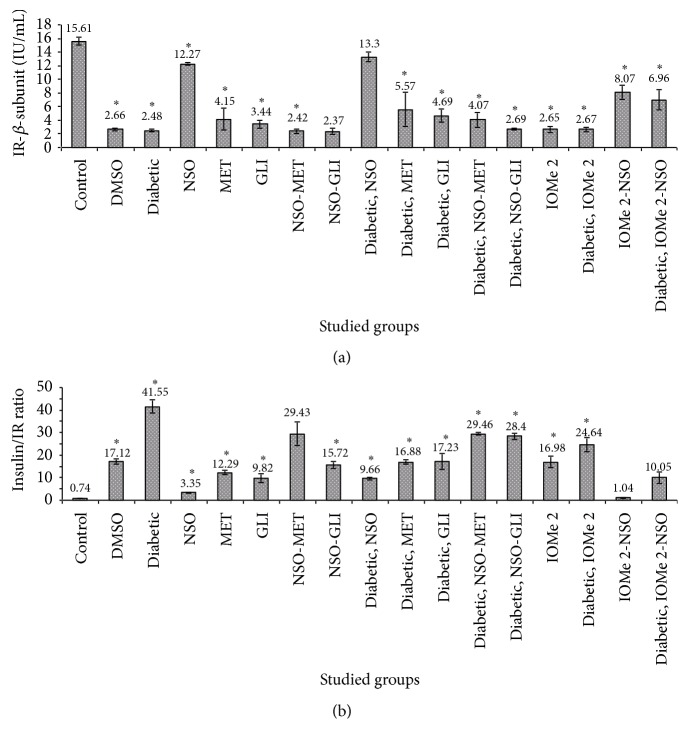
Levels of IR *β* subunit (a) and insulin/IR ratio (b) in rat sera after 21 days of the treatment with NSO, reference drugs, and I-OMe-AG538. Data are expressed as mean ± SE. Significance is shown as (*∗*) at *p* < 0.05,  *n* = 7.

**Figure 4:**
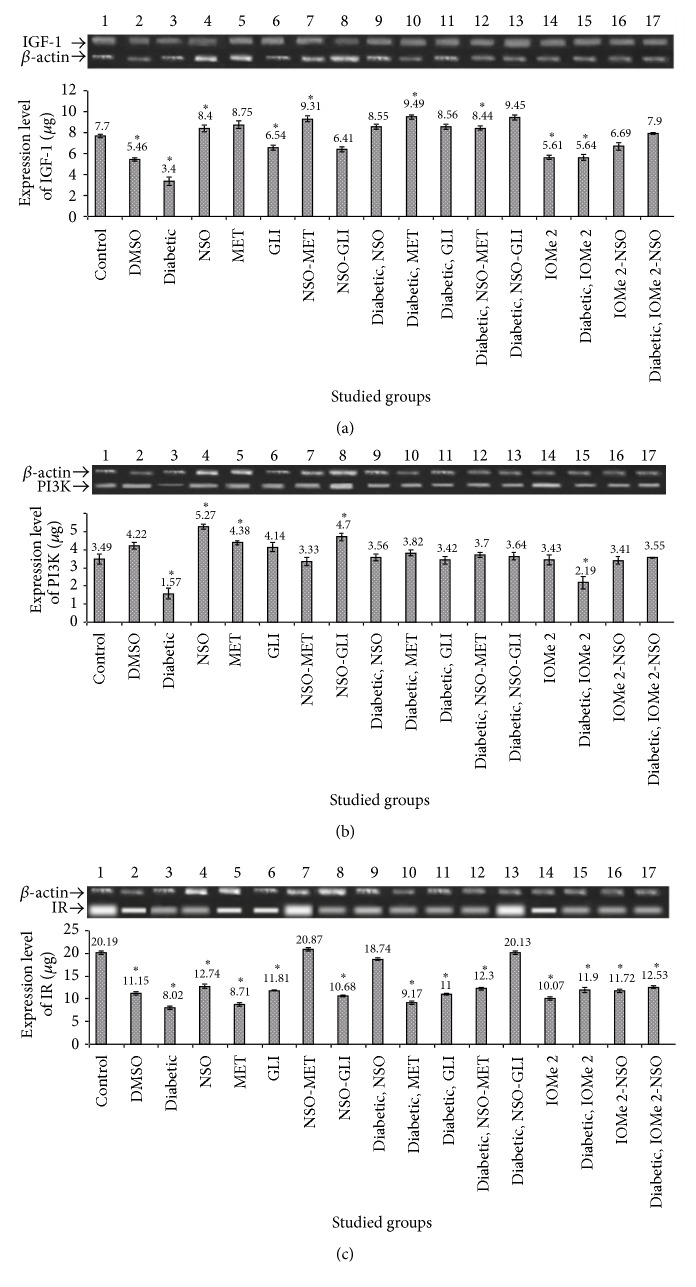
Effect of NSO, reference drugs, and I-OMe-AG538 on the gene expression ratio of IGF-1/*β*-actin (a), PI3K/*β*-actin (b), and IR/*β*-actin (c) in rats of all studied groups. The insets show agarose gel electrophoresis of gene expression of IGF-1 (572 bp), PI3K (101 bp), and IR (129 bp) compared to *β*-actin (300 bp) (data are expressed as mean ± SE, *n* = 3). ^*∗*^Significant at *p* < 0.05.

IGF-1, PI3K, and IR gene expressions were decreased (3.9-, 2.4-, and 1.8-fold, resp.), whereas the IR *β*-subunit level was decreased. The ratio of insulin/IR *β*-subunit was increased in diabetic rats compared to normal control (Figures 3(a) and 3(b)). Also, DMSO-administrated group showed a reduction of PI3K and IR gene expressions (2.2- and 1.8-fold, resp.), an elevation of the ratio of insulin/insulin receptor *β*-subunit (23.14-fold), and an increase of IGF-1 gene expression (4.9-fold) compared to normal control. The treatment of diabetic rats with NSO and NSO-GLI leads to more gene expressions of IGF-1 (5.6- and 11.3-fold, resp.), PI3K (2.4- and 5.9-fold, resp.), and IR (1.6- and 1.8-fold, resp.) (Figures 4(a)–4(c)). A reduction of insulin/IR ratio (4.3- and 1.5-fold, resp.) compared to diabetic rats was noticed (Figure 3). However, the treatment of diabetic rats with MET and GLI did not show any effect on gene expressions. These drugs just caused a decrease of the ratio of insulin/IR (2.5- and 2.4-fold, resp.) compared to diabetic rats (Figure 3(b)). Injection of I-OMe-AG538 leads to (3.9-, 2.1-, and 2-fold decrease, resp.) in expression of IGF-1, PI3K, and IR expressions and (23.0-fold increase) in ratio of insulin/IR compared to normal control (Figures 3(b), 4(a), 4(b) and 4(c)). Also, diabetic rats injected with I-OMe-AG538 showed a reduction in expression of IGF-1, PI3K, and IR (5.6-, 2.2-, and 1.8-fold, resp.) and an increase in the ratio of insulin/IR (33.3-fold) compared to normal control. Administration of NSO to I-OMe-AG538-injected nondiabetic group leads to an increase of the expression of IGF-1 and PI3K (8.8- and 4.9-fold, resp.) and a decrease in the ratio of insulin/IR (16.3-fold) compared to I-OMe-AG538-injected nondiabetic group. Also, NSO administration to I-OMe-AG538-injected diabetic group showed an increase of the expression of IGF-1, PI3K, and IR (12.3-, 5.1-, and 1.4-fold, resp.) compared to I-OMe-AG538-injected diabetic group (Figures 4(a)–4(c)).

### 3.6. Inflammatory Parameters

**Figure 5:**
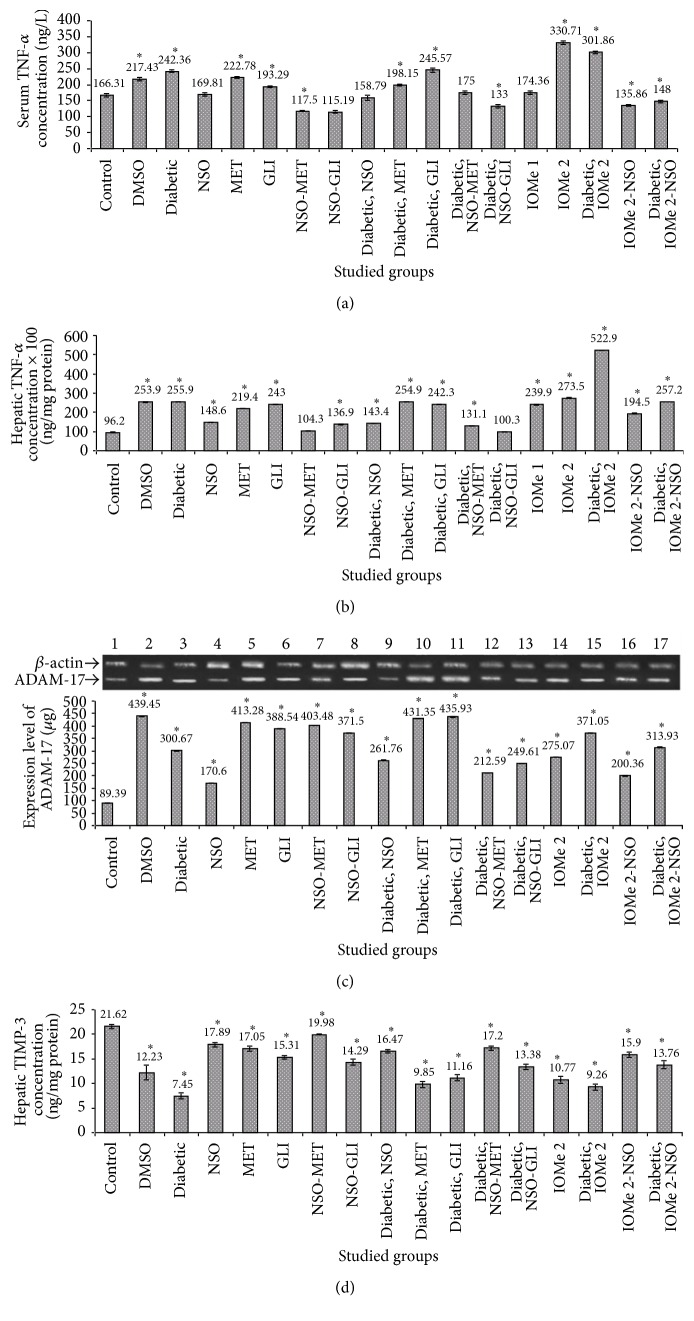
Effect of NSO, reference drugs, and I-OMe-AG538 on serum (a) or hepatic (b) TNF-*α* levels, gene expression ratio of ADAM-17/*β*-actin (c), and protein expressiom of TIMP-3 (d) in rats of all studied groups. The inset in (c) shows agarose gel electrophoresis of gene expression of ADAM-17 (157 bp) compared to *β*-actin (300 bp) (data are expressed as mean ± SE for 21 days of treatment, *n* = 7). ^*∗*^Significant at *p* < 0.05.

Serum and hepatic TNF-*α* levels were increased in the diabetic group (1.5- and 2.7-fold, resp.) and in the DMSO group (1.3- and 2.6-fold, resp.) compared to normal control (Figures 5(a) and 5(b)). Also, ADAM-17 expression was increased (1.3- and 4.1-fold, resp., in diabetic and DMSO groups) compared to normal control (Figure 5(c)). The treatment of diabetic rats for 21 days with NSO, NSO-MET, and NSO-GLI revealed amelioration of serum TNF-*α* level (1.5-, 1.2-, 1.4-, and 1.8-fold, resp.) compared to diabetic group. Also, the treatment of diabetic rats with MET leads to a reduction of only serum TNF-*α* level (1.2-fold) compared to the diabetic group. ADAM-17 expression was reduced only after the treatment of diabetic rats with GLI (1.3-fold decrease) compared to diabetic rats. Moreover, serum and hepatic TNF-*α* levels were increased in both I-OMe-AG538-injected group (2- and 2.8-fold, increase resp.) and I-OMe-AG538-injected diabetic group (1.8- and 5.4-fold, resp.) compared to normal control. Administration of NSO to I-OMe-AG538-injected nondiabetic group leads to a reduction of serum and hepatic TNF-*α* levels (2.4- and 1.4-fold, resp.). Also, the administration of NSO for the diabetic group injected with I-OMe-AG538 leads to 2.0-fold decrease of both serum and hepatic TNF-*α* levels. ADAM-17 expression was increased (3.1- and 3.7-fold) in I-OMe-AG538-injected nondiabetic and diabetic groups, respectively, compared to normal control. Administration of NSO to I-OMe-AG538-injected nondiabetic and diabetic groups reduces ADAM-17 expression by 1.3- and 1.5-fold, respectively (Figures 5(a)–5(c)). The obtained data of ADAM-17 were confirmed by the results of TIMP-3 expression as reversing changes of TIMP-3 to those of ADAM-17 were detected (Figure 5(d)).

### 3.7. Histological Studies

**Figure 6:**
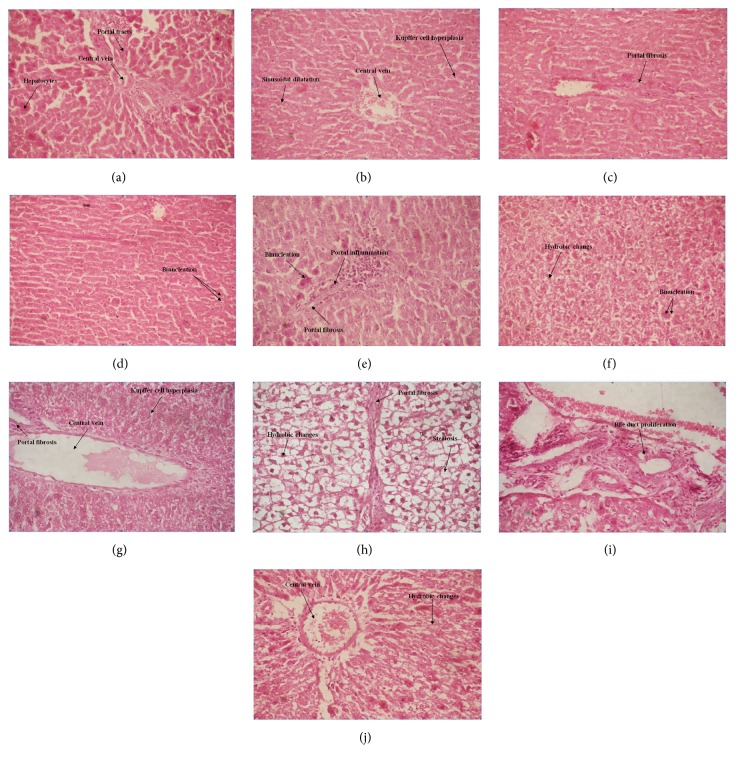
Photographs of rat hepatocytes sections in the different experimental groups, stained with hematoxylin and eosin. (a) Control rats; (b–d) diabetic rats; (e) NSO-treated diabetic rats; (f–h) IOMe 2-injected normal rats; (i-j) IOMe 2-injected diabetic rats (x: 400).

The biochemical alterations were confirmed by the histological studies (Figures 6(a)–6(j)). In the panels of Figure 6, control rat liver revealed normal hepatocytes with central vein and portal tract (a), whereas diabetic rat liver revealed central vein congestion, kupffer cell hyperplasia, and sinusoidal dilatation (b). Diabetic rat liver revealed moderate portal fibrosis (c) and binucleation of the hepatocytes (d). Diabetic rat liver treated with NSO for 21 days revealed binucleation of hepatocytes, moderate portal inflammation, and moderate portal fibrosis (e). Normal rats injected with IOMe 2 for 21 days revealed hydropic changes with binucleation of the hepatocytes (f), whereas central vein congestion with kupffer cell hyperplasia and mild portal fibrosis (g) were shown. Also, this group showed hydropic changes in hepatocytes with steatosis and portal fibrosis (h). The diabetic rat liver injected with IOMe 2 for 21 days showed bile duct proliferation (i), central vein congestion, and hydropic changes in hepatocytes (j).

## 4. Discussion

The current study was initiated with the objective of developing an ideal animal model for type II diabetes. The study provided further insight into the roles of NSO alone or in combination with MET or GLI in type II diabetes mellitus. This may help to set a new direction toward the development of effective treatment. Also, the mechanism of action of I-OMe-AG538 was investigated. As explained in the above-mentioned results, hyperinsulinemia was detected in the diabetic rats and that may be due to selected destruction of pancreatic islet cells. So some cells still survive and insulin secretion can be stimulated in the residual *β*-cells of these diabetic rats. The occurrence of insulin resistance was confirmed by elevation of HOMA-IR to 67.5-fold and reduction of HOMA-*β* to 4.7%. In insulin resistant state, insulin is unable to act properly on resistant tissues and this results in poor glucose utilization. Therefore, *β*-cells initially compensated for insulin resistance by increasing insulin secretion [36]. Also, the unesterified lipids interfere with the IR-insulin interaction by reducing the affinity of IRs for insulin. Thus, the reduction of glucose uptake by the body cells consequently leads to hyperinsulinemia [37]. Furthermore, saturated fatty acids cause change in the composition of plasma membrane fatty acid, impaired insulin signaling, and reduced insulin sensitivity [38]. Another theory indicated that neither defects in IR function nor elevated membrane glycoprotein PC-1 activities are involved in the development of insulin resistance in rat with HFD feeding. Such insulin resistance is likely due to a postreceptor defect in skeletal muscles [39].

There is another symptom of the insulin resistance that is an abnormal blood lipid profile. The current study showed increases in TC, TG, LDL-c, and AI and decrease in HDL-c. These changes are attributed to increased flux of free fatty acids into the liver secondary to insulin deficiency or insulin resistance [40], which lead to excess fatty acid accumulation in the liver and converted to TG [41]. The impaired ability of insulin to inhibit free fatty acids release leads to an elevated hepatic VLDL-c production [42]. The elevation of VLDL-c and TG levels leads to a decreased level of HDL-c and an increased concentration of small LDL-c particles by activation of lipoprotein lipase and lecithin acyl-cholesterol transferase [43]. Lipid profile changes in STZ-induced HFD diabetic rats maybe also occur as a result of obesity [44]. Hypercholesterolemia in STZ-induced HFD diabetic rats may be due to an increased dietary cholesterol absorption from the small intestine following the intake of HFD in the diabetic condition [45]. Also, it may be attributed to the inhibition of cholesterol catabolism [46]. Hypertriglyceridemia in case of diabetes mellitus and insulin resistance may result from the accumulation of very low density lipoparticles by either overproduction or decreased catabolism or both. The inability of the fat cell adequately to store excess TG is a likely first step in the underlying hypertriglyceridemia of insulin resistance [17]. Also, hypertriglyceridemia may be attributed to an increased absorption and formation of TG in the form of chylomicrons following exogenous consumption of fat-rich diet or through the increased endogenous production of TG enriched hepatic VLDL-c and decreased TG in peripheral tissues [16]. However, it was reported that oral administration of NS extracts to diabetic rats showed significant decrease in the plasma lipid profile level [47]. Treatment of rats with NS seeds causing hypocholesterolemic effect was also reported [48].

In diabetes, some insulin signaling pathways may be disrupted. Insulin resistance is partly mediated by reducing levels of IR expression which means downregulation [49]. This leads to impaired tyrosine phosphorylation of IR and subsequent tyrosine phosphorylation of IRS-1 and the association of P85/PI3K activity with IRS-1 in response to insulin was attenuated. In contrast to other growth factors, IR does not bind to PI3K directly but uses rather docking protein such as IRS proteins. All of these lead to inhibition of the association with P85 regulatory subunit of PI3K and subsequent deactivation of P110 catalytic subunit [50]. The current study showed 3.9-fold decrease in IGF-1 expression in the diabetic group compared to control group, which may be independently associated with obesity, glucose intolerance, diabetes, and atherogenic dyslipidemia [51]. IGF-1 reduction causes an interruption of the interaction with tyrosine kinase membrane receptor linked to IRS-1 and IRS-2. Therefore, when the reduction of PI3K signaling pathway occurs, Akt will be activated and hence GLUT4 vesicle translocation and reduction of glucose transport will occur [52]. As a result of abnormal lipid metabolism and an elevation of lipid deposition in the skeletal muscles during the insulin resistance condition, insulin activity impaired, plasma free fatty acids increased, and insulin-stimulated glucose uptake reduced [53]. The findings in the above-mentioned results are inconsistent with other reports which clearly demonstrated that IR signaling through PI3K pathway is reduced in the rat model of insulin resistance [54].

There is growing evidence that an increased release of proinflammatory cytokines is associated with the development of insulin resistance [55]. The stimulated TNF-*α* production contributes to *β*-cell degradation [56]. Moreover, hyperglycemia increases matrix metalloproteinases and ADAM activities, which may be linked to unbalanced expression of TIMP-3 [57]. Therefore, the reduction of TIPM3 results in increased ADAM-17 and TNF-*α* [58]. In addition, the reduction of antioxidants and elevation of ROS in diabetes mellitus lead to stimulation of TNF-*α* and ADAM-17 production. It is reported that there is a relationship between TNF-*α* increase in diabetes and impairment of insulin signaling pathway by increasing serine phosphorylation of IRS-1 which inhibits IR tyrosine kinase activity and leads to downstream signaling [59]. The increase of serum and hepatic TNF-*α* in diabetic rats in the present study comes in accordance with other reported studies [60, 61]. Also, ADAM-17 expression in hepatic tissue of diabetic rats is 1.3-fold increase and this agreed with previous studies [62].

In the current study, two doses of the tyrphostin I-OMe-AG538 were broadly investigated. It should be mentioned that the subjected doses used are mainly in the range between 1/1000 and 1/100 LD_50_, which suppose comparable results. An elevation of blood glucose, cholesterol, and TG was detected in I-OMe-AG538-treated group. It was reported that HOMA-IR was increased during insulin resistance, while HOMA-*β* was decreased, which indicates the action of I-OMe-AG538 on IR [63]. From the biochemical background, I-OMe-AG538 makes blocking of the insulin to bind with the receptor and the increased insulin leads to a condition similar to type I diabetes mellitus. With long time of treatment of the rats with I-OMe-AG538 condition of insulin resistance occurred and *β*-cell function was decreased and it was not able to perform its function. So, the current study showed in diabetic group which injected I-OMe-AG538 blood glucose level was decreased compared to control diabetic rats.

In fact, it was reported that treatment with NS extract induced sensitization in rat hepatocytes to the action of insulin by enhancing the activity of two major intracellular signal transduction pathways of IR [64]. Consequentially, insulin resistance could be decreased at target tissues by the same mechanisms. The mechanism of improved tissue sensitivity to insulin action by NS may be related also to the reduction in oxidative stress [65, 66]. In the current study, it is demonstrated that NSO plays an important role in improving of insulin signaling pathway of diabetic rats. Both IGF-1 and PI3K expressions were increased by 5.5- and 2.4 fold, respectively. This affects the signaling molecule Akt that activates GLUT4 and GLUT4 is then translocated to the membrane and imports glucose into the cell [67].

The interaction of herbs with drugs is well known. Most of the diabetic patients use the various antidiabetic herbs along with antidiabetic drugs for controlling their blood glucose level [68]. The current study showed a significant reduction of blood glucose, insulin, HOMA-IR, TC, AAI, TBARS, NO, and TNF-*α* levels and insulin/IR ratio of diabetic rats given NSO-GLI compared to diabetic rats given NSO alone. Moreover, diabetic rats given combination of NSO-MET showed a significant reduction of insulin, HOMA-IR, IR, TG, AAI, TBARS, NO, and AST levels compared to diabetic rats given NSO alone. These results may be attributed to the effect of herbal drug interaction, which may be pharmacodynamic or pharmacokinetic in nature. Pharmacodynamic interaction occurs when herbs exert either synergistic or antagonistic effect with conventional drug [69]. Most of the diabetic patients use the various antidiabetic herbs along with antidiabetic drugs for controlling their blood glucose level [68]. Hence, it could be suggested that there may be a chance of interaction between herbs and drug. In conclusion, the obtained data of the present study support the antidiabetic effect of NSO through a linked investigation of lipid profile, antioxidant activity, and signaling molecules in the absence and presence of some antidiabetic drugs. This suggests that there may be a chance of interaction between herbs and drug.

## Figures and Tables

**Table 1:** Composition of HFD.

Ingredients	Diet (g/kg)
Powdered NPD	365
Butter	310
Casein	253
Cholesterol	10
Vitamins and mineral mix	60
Yeast powder	1
Sodium chloride	1

**Table 2:** PCR primers and programs of PCR cycle.

Gene	Forward primer	Reverse primer	PCR program
*β*-actin	5′-CTGACCGAGCTGGCTAC-3′	5′-CCTGCTTGCTGATCCACA-3′	94/2/53.4/30/72/30/30
ADAM-17	5′-TAGCAGATGCTGGTCATGTG-3′	5′-TTGCACCACAGGTCAAAAG-3′	94/2/50/30/72/30/30
PI3K	5′-TTAAACGCGAAGGCAACGA-3′	5′-CAGTCTCCTCCTGCTGTCGAT-3′	94/2/53.7/30/72/30/30
IGF-1	5′-ACTTCTGCGCCAACATCCTCA-3′	5′-CCCTTTAGTCCCCGTCACTTCC-3′	94/2/57/30/72/30/30
IR	5′-TGACAATGAGGAATGTGGGGAC-3′	5′-GGGCAAACTTTCTGACAATGACTG-3′	94/2/53.3/30/72/30/30

**Table 3:** BGL, AI, AAI, insulin, HOMA-IR, and HOMA-*β* values after 21-day treatment with NSO, reference drugs, and I-OMe-AG538. Values are expressed as mean ± SE (*n* = 10). Significance: ^*∗*^ *p* < 0.05, ^*∗∗*^ *p* < 0.01, and ^*∗∗∗*^ *p* < 0.001 compared to control.

Studied groups	BGL (mg/dL)	AI	AAI (%)	Insulin (*µ*IU/mL)	HOMA-IR (mg/dL)	HOMA-*β* (%)
Control	75.53 ± 1.22	1.70 ± 0.06	142.63 ± 20.08	11.59 ± 1.76	2.16 ± 0.89	33.299 ± 0.908
DMSO	116.07 ± 2.66^*∗∗∗*^	1.42 ± 0.04	138.58 ± 2.25	45.00 ± 0.68^*∗∗∗*^	12.90 ± 0.76^*∗∗∗*^	30.526 ± 1.301^*∗∗*^
Diabetic	581.31 ± 36.31^*∗∗∗*^	16.82 ± 1.25^*∗∗∗*^	6.32 ± 0.56^*∗∗∗*^	101.59 ± 5.78^*∗∗∗*^	145.82 ± 1.43^*∗∗∗*^	7.056 ± 0.512^*∗∗∗*^
NSO	95.70 ± 1.89^*∗∗∗*^	1.15 ± 0.30	676.37 ± 37.56^*∗∗∗*^	41.10 ± 1.87^*∗∗∗*^	9.71 ± 1.25^*∗∗∗*^	45.248 ± 0.456^*∗∗∗*^
MET	56.37 ± 0.61^*∗∗∗*^	1.73 ± 0.22	137.40 ± 12.45	30.34 ± 1.26^*∗∗∗*^	4.22 ± 0.67^*∗*^	−164.742 ± 0.636^*∗∗∗*^
GLI	57.62 ± 1.46^*∗∗∗*^	1.71 ± 0.09	141.03 ± 9.08	31.77 ± 1.42^*∗∗∗*^	4.52 ± 0.45^*∗*^	−212.587 ± 0.123^*∗∗∗*^
NSO-MET	101.67 ± 3.05^*∗∗∗*^	1.56 ± 0.36	179.13 ± 15.06^*∗*^	37.73 ± 0.60^*∗∗∗*^	9.47 ± 0.83^*∗∗*^	35.125 ± 1.086^*∗∗*^
NSO-GLI	110.16 ± 1.84^*∗∗∗*^	1.65 ± 0.19	153.55 ± 10.12	36.44 ± 0.75^*∗∗∗*^	9.91 ± 1.26^*∗∗*^	27.817 ± 0.978^*∗∗∗*^
Diabetic, NSO	142.76 ± 16.94^*∗∗*^	1.00 ± 0.06	23147.62 ± 42.96^*∗∗∗*^	127.86 ± 1.27^*∗∗∗*^	45.07 ± 0.66^*∗∗∗*^	57.710 ± 0.805^*∗∗∗*^
Diabetic, MET	265.23 ± 20.67^*∗∗∗*^	5.72 ± 1.45^*∗*^	21.18 ± 1.08^*∗∗∗*^	54.03 ± 2.45^*∗∗∗*^	35.42 ± 0.56^*∗∗∗*^	9.618 ± 0.526^*∗∗∗*^
Diabetic, GLI	359.94 ± 17.29^*∗∗∗*^	3.78 ± 1.26^*∗*^	35.96 ± 8.45^*∗∗∗*^	74.47 ± 5.39^*∗∗∗*^	66.18 ± 1.47^*∗∗∗*^	9.028 ± 0.538^*∗∗∗*^
Diabetic, NSO-MET	112.46 ± 3.94^*∗∗∗*^	1.42 ± 0.05	240.83 ± 16.85^*∗∗∗*^	90.80 ± 2.70^*∗∗∗*^	25.21 ± 0.98^*∗∗∗*^	66.09 ± 1.123^*∗∗∗*^
Diabetic, NSO-GLI	106.62 ± 3.79^*∗∗∗*^	1.41 ± 0.89	245.85 ± 8.03^*∗∗∗*^	76.11 ± 0.86^*∗∗∗*^	20.04 ± 0.89^*∗∗∗*^	62.814 ± 0.907^*∗∗∗*^
IOMe 2	124.59 ± 3.26^*∗∗∗*^	1.73 ± 0.30	137.89 ± 6.02	42.80 ± 1.18^*∗∗∗*^	13.17 ± 0.45^*∗∗∗*^	25.017 ± 0.625^*∗∗∗*^
Diabetic, IOMe 2	224.62 ± 4.29^*∗∗∗*^	12.54 ± 2.85^*∗∗∗*^	8.67 ± 1.25^*∗∗∗*^	63.54 ± 3.38^*∗∗∗*^	35.24 ± 0.47^*∗∗∗*^	14.153 ± 0.925^*∗∗∗*^
IOMe 2-NSO	94.26 ± 1.43^*∗∗∗*^	1.42 ± 0.09	236.49 ± 4.56^*∗∗∗*^	5.49 ± 2.16^*∗∗∗*^	1.28 ± 0.56	6.322 ± 0.347^*∗∗∗*^
Diabetic, IOMe 2-NSO	113.54 ± 1.83^*∗∗∗*^	1.52 ± 0.35	191.68 ± 8.47^*∗∗*^	62.56 ± 1.59^*∗∗∗*^	17.54 ± 1.49^*∗∗∗*^	44.562 ± 0.908^*∗∗∗*^

## Abbreviations

DM: Diabetes mellitus
STZ: Streptozotocin
NPD: Normal pellet diet
HFD: High-fat diet
NSO: * Nigella sativa* oil
MET: Metformin
GLI: Glimepiride
NSO-MET: Combination of NSO and MET
NSO-GLI: Combination of NSO and GLI
TC: Total cholesterol
TG: Triacylglycerol
LDL-c: Low density lipoprotein cholesterol
HDL-c: High density lipoprotein cholesterol
HOMA-IR: Homeostatic model assessment of insulin resistance
HOMA-*β*: Homeostatic model assessment of *β*-cell function
AI: Atherogenic index
AAI: Antiatherogenic index
TIMP-3: Tissue inhibitor of metalloproteinase 3
IR: Insulin receptor
IRI: Insulin receptor inhibitor
IGF-1: Insulin-like growth factor-1
PI3K: Phosphoinositide 3 kinase
ROS: Reactive oxygen species
BGL: Blood glucose level
TBARS: Thiobarbituric acid reactive substances
RT-PCR: Real-time polymerase chain reaction
DEPC: Diethylpyrocarbonate
RT: Reverse transcriptase.
